# Moth-eye shaped on-demand broadband and switchable perfect absorbers based on vanadium dioxide

**DOI:** 10.1038/s41598-020-59729-2

**Published:** 2020-03-11

**Authors:** Trevon Badloe, Inki Kim, Junsuk Rho

**Affiliations:** 1 Department of Mechanical Engineering, Pohang University of Science and Technology (POSTECH), Pohang, 37673 Republic of Korea; 2 Department of Chemical Engineering, Pohang University of Science and Technology (POSTECH), Pohang, 37673 Republic of Korea

**Keywords:** Metamaterials, Nanophotonics and plasmonics, Information technology, Computer science

## Abstract

Two biomimetic, moth-eye structure, perfect absorbers in the visible and near infrared regions are introduced and investigated. The moth-eye structure is made up of vanadium oxide (VO_2_), which is a phase change material that changes from an insulator state to a metallic state at around 85 °C. The VO_2_ structure sits on top of a sapphire (Al_2_O_3_) dielectric spacer layer, above a gold (Au) back reflector. Two perfect absorbers are designed, one with perfect absorption over an ultra-broadband range between 400 and 1,600 nm, for both the insulating and metallic phases, while the second can switch between being a perfect absorber or not in the range 1,000 and 1,600 nm. The absorption profiles and electric and magnetic fields are examined and discussed to provide insight into how absorbers function in the four different situations.

## Introduction

Metamaterials made of subwavelength structures that interact with light in ways that cannot be found in nature have been investigated widely in recent years. In particular, absorption has been studied for various applications such as, solar photovoltaics^1^, infrared camouflage^2^ and radiative cooling^3^. There are various designs of perfect absorbers that can create either broadband or narrowband responses, each of which have their own pros and cons for any given application. The materials are often structured and designed in a way to cause resonances between the structures and the incoming electromagnetic field, thereby confining and absorbing it^4^. Techniques to achieve this include using nanoantenna^5,6^, multilayer Fabry-Perot resonator thin films^7^ and nanostructured metasurfaces^8–11^.

The eyes of moths are made up of an array of antireflective structures. They contain parabolic nano-hemispheres with dimensions smaller than that of visible light that act as a region of graded refractive index between the ambient medium and the interface. This natural feature is helpful for the moths as a mechanism to avoid any unwanted reflection of light and detection from predators. Moths eyes have been studied theoretically and fabricated experimentally to display their antireflective properties, and we previously designed an ultra-broadband perfect absorber based on this shape using a range of different transition metals^12^. The biggest downside of current metamaterials is that after a device has been designed and fabricated, it can only work in a given range or for the one specific use it was designed for. To develop metamaterials into real-life, functional devices, research into active metamaterials has recently started. Some examples of active metamaterials use liquid crystals to change the refractive index of the surrounding medium^13^, flexible substrates to manipulate the resonances between meta-atoms^14^, and phase change materials such as Ge_2_Sb_2_Te_5_ (GST) and Sb_2_S_3_^15–19^ that give different responses in different phases.

Phase change materials (PCM) have extremely useful characteristics for active nanophotonics. An external stimulus such as heat, light or stress causes a PCM to change phase, from a crystalline to amorphous state, or from a metallic to an insulating state, and vice versa. The switching between phases is usually fast and stable over numerous changes and has the advantage of reversibility between the two states. They have been employed in applications such as, radiative cooling^20^, data storage^21,22^, and even in textiles^23^.

Here, we use vanadium dioxide (VO_2_) as the material for moth-eye structures due to its interesting temperature dependent phase transition between an insulator and a metal that occurs at around 85 °C. VO_2_ is a more practical material as it is a ‘volatile’ PCM, whereas GST and Sb_2_S_3_ are ‘non-volatile’. That is to say, the phase of VO_2_ depends entirely on if the external stimulus is being applied or not, whereas the others state is maintained even after the stimulus has been removed. This allows the phase of VO_2_ to be controlled simply by its temperature, without having to apply some form of rapid heating or cooling to change its state.

**Figure 1:**
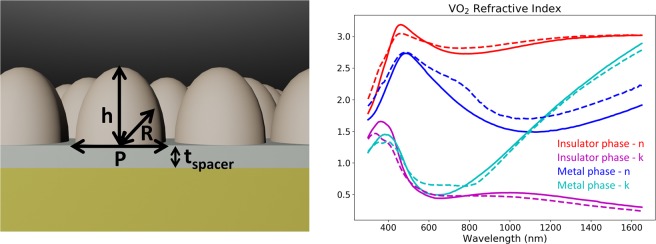
(**a**) A schematic of the parabolic VO_2_ moth-eye structures on an Al_2_O_3_ spacer, over an Au back reflector. (**b**) The measured refractive index (real (n) and imaginary (k) parts) for the metallic and insulating phases of VO_2_. Solid lines represent VO_2_ sputtered on an Al_2_O_3_ substrate, while dashed lines represent VO_2_ sputtered on an Si substrate.

We place the VO_2_ moth-eye structures on top of a sapphire (Al_2_O_3_) spacer layer, above a gold (Au) back reflector to construct a broadband perfect absorber, a schematic is shown in Fig. 1a. By optimising the periodicity (P) of the unit cell of a hexagonal lattice, the height (h) and the radius of curvature (c), we designed two types of broadband perfect absorbers. The first, an ultra-broadband perfect absorber with ~99.6% absorption from 400 to 1,600 nm in both the metallic and insulator phases, and the second, a switchable broadband absorber in the near infrared (NIR) range, that can switch between an almost perfect absorber with absorption of ~98.7%, to a poor absorber of ~40% when switching from the metallic to insulating phase. As VO_2_ undergoes its phase transition from an insulating phase at low temperatures, to a metallic phase at higher temperatures, its properties in the visible regime are largely unchanged, whereas the imaginary part of its refractive index, i.e. absorption, at near infrared wavelengths increases dramatically. The VO_2_ film samples were prepared using the sputtering method, followed by an annealing process. This allows us to produce a very stable VO_2_ film that can does not oxidise instantly. We produced samples on both a silicon (Si), and an Al_2_O_3_ substrate. Their temperature dependent refractive index was measured with a temperature-controlled ellipsometer. The refractive index of the VO_2_ is very similar regardless of substrate, owing to the sputtering method used to grow the film. This allows us to produce a VO_2_ film that has a consistent refractive index, regardless of the substrate or thin film it is grown on. The measured refractive indexes are shown in Fig. 1b, and the simulations in this manuscript were performed using the refractive index from the VO_2_ on an Al_2_O_3_ substrate. The phase switching properties of VO_2_ have been used previously to design switchable absorbers by several other research groups, but these devices usually operate in the THz regime^24–26^, or are polarisation dependent^27,28^. Our design operates in the ultra-broadband region from visible to NIR (400–1,600 nm) and is largely polarisation independent. The devices designed here are suitable for applications where the operating temperature plays a role in if we want absorption, or where the operating temperature can be manually controlled. This near perfect absorption is essentially incident angle insensitive for up to 60 degrees, for both TE and TM mode illumination. The influences of the metal reflector, dielectric spacer thickness, and the working mechanisms of the broadband perfect absorbers are explored and discussed below.

## Results and Discussion

We define the surface of the parabolic moth-eye shape by ${\rm{S}}=\frac{{\rm{c}}{r}^{2}}{2}.$ The values c = 1/R, and ${r}^{2}={x}^{2}+{y}^{2}$ are the inverse of the radius of curvature and the position on the surface respectively. The periodicity of the hexagonal unit cell is defined as P, while the height of the moth-eye structure is given as h and the thickness of the dielectric spacer layer is t_spacer_.

### Ultra-broadband perfect absorber

**Figure 2:**
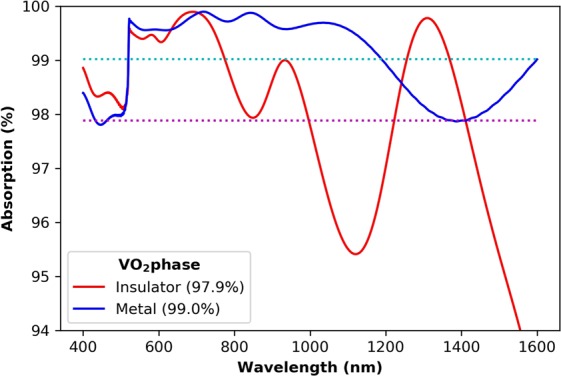
Absorption spectra in the metallic and insulating phases for the moth-eye structured perfect absorber.

The first design produces an ultra-broadband perfect absorber from 400 to 1,600 nm, with a periodicity, P, of 600 nm, height, h, of 700 nm, dielectric spacer layer thickness, t_spacer_, of 100 nm, and radius of curvature, R, of 56 nm. At low temperatures, i.e. when the VO_2_ is in the insulator phase, the moth-eye structured absorber achieves an average of 97.9% absorption across the ultra-broadband range. At higher temperatures, when the VO_2_ has changed to its metal phase, the absorption increases slightly to an average of 99.0% over the same span, as shown in Fig. 2.

**Figure 3:**
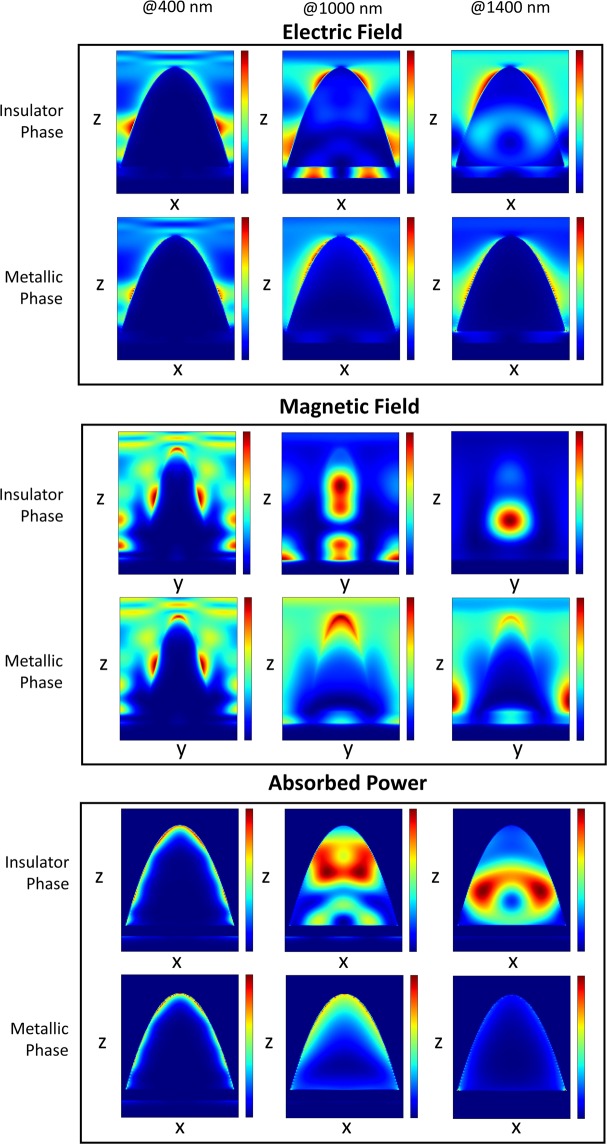
The normalised field profiles for (**a**) the electric and (**b**) magnetic fields, and (**c**) the absorbed power at selected wavelengths, chosen to represent where the fields have interesting and differing features.

The plot shows that the perfect absorber has a very similar response in the visible regime, from 400 to around 800 nm, for both phases of VO_2_. In this region, the refractive index and extinction coefficients of both states are comparable. Meanwhile. in the near IR region, we can see some differences due to the metallic phase VO_2_ having an increased absorption coefficient and a lower refractive index. Despite this, the designed structure still exhibits perfect absorption over the same, ultra-broadband range. To investigate the absorption mechanisms, we plot the normalised calculated electric and magnetic fields, and the absorbed power distributions in Fig. 3.

As we can see from the field profiles in Fig. 3, at visible wavelengths, the moth-eye absorber produces very similar profiles for both phases of VO_2_. At 1,000 nm, the electric field for the insulating phase starts to be confined in the dielectric layer between the structure and back reflector, while the magnetic field starts to form dipole resonances inside the structures. In the case of the metallic phase, the electric cannot penetrate the structure, so it is confined between the structures, while the magnetic fields form resonances between the tops of the structures and the back reflector, then at longer wavelengths the magnetic field gets strongly confined between the gaps in the structures. We can see that in the insulating phase, at infrared wavelengths, the power is mostly absorbed inside the structure in the magnetic field, which can be attributed to Mie-like resonances since the wavelength inside the structures is comparable to IR wavelengths. Meanwhile in the metallic phase, the power is lost in the gaps between the structures, above the dielectric spacer layer. This can be attributed to the excitation of localised surface plasmons in the small gaps. These small gaps are an indicative feature of designing moth-eye structure absorbers^29^, as the anti-reflective performance is increased since there is less area with an abrupt refractive index change. The dielectric spacer layer thickness plays no role in these mechanisms, so it has little effect on the ultra-broadband absorption for this design.

The parabolic shape of moth-eye structures creates a gradient in the refractive index, which creates a broadband impedance matching effect^30^. Since the Au back reflector restricts the transmission to zero, the aforementioned resonances combined with the broadband impedance matching conditions that limit reflection, allow for ultra-broadband absorption. In the view of fabrication, a parabolic shape is fairly difficult to achieve, so we investigated four other shapes, namely a cone, square based pyramid, a circular rod, and a square rod, to see how the shape of the structure affects the absorption. To do this, we used the same size shape for the base (in the case of the cone and rod), while for square based structures we used a square of sides equal to the radius of the circular base. The results are presented in Table 1. Since the design was optimised for the moth-eye shape, it unsurprisingly shows the best results. The cone and pyramid shapes show fairly similar performance to the moth-eye shape, especially in the metallic phase. While the linearly tapered shape of the cone and pyramid creates a form of gradient in the refractive index, the magnetic field confinement inside the structure is ultimately reduced. The circular and square rods have a consistent refractive index across their entire structure which inhibits their broadband absorption capability in the metallic phase, as the structure does not act as an impedance matching anti-reflective layer.

**Table 1:** The average broadband absorption (from 400–1,600 nm) for different shaped VO_2_ structures on an Al_2_O_3_ spacer, on top of an Au back reflector.

	Moth-eye	Cone	Pyramid	Circular Rod	Square Rod
Insulator Phase	97.9%	89.2%	88.1%	87.7%	83.5%
Metal Phase	99.0%	98.8%	99.0%	84.6%	82.1%

**Figure 4:**
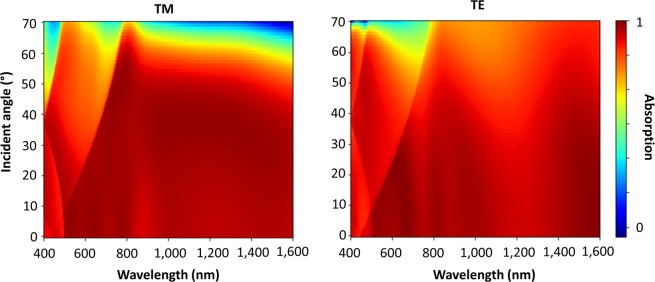
The angular response of the moth-eye broadband absorbers for (**a**) TM and (**b**) TE polarised incident light.

Figure 4 shows the angular dependence of the VO_2_ moth-eye absorber, for both TE and TM polarisation. It is extremely robust to the angle of incidence with absorption over 90% for incident angles of up to 70 degrees, and over 95% up to 60 degrees for both polarisations. This absorber is particularly robust for TE polarised light in the IR region, and still produces peaks of perfect absorption at large incident angles for TM polarisation. The slight differences at higher angles are due to the electric field rotating out of the plane of the moth eye structures, thereby limiting the amount of possible confinement.

### Switchable absorber

**Figure 5:**
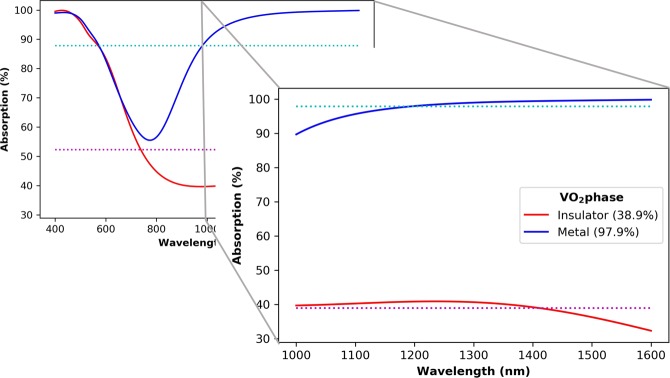
The absorption profiles of the switchable absorber. At IR wavelengths the response changes from an almost perfect absorber to a poorly absorbing structure as the phase changes from a metallic to insulator.

The second absorber that we report here has P = 200 nm, h = 200 nm, t_spacer_ = 100 nm, and R = 20 nm. This is considerably smaller than the first design and creates a switchable absorber in the near IR region. By switching between the insulator and metallic phases, the average absorption from 1,000 to 1,600 nm changes from a non-absorbing 38.9% to almost perfect absorption of 97.9% as shown in Fig. 5.

**Figure 6:**
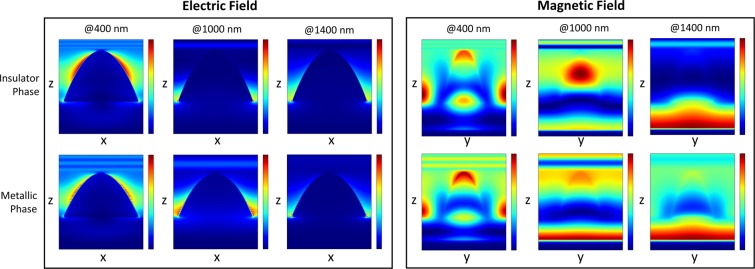
The field profiles for (**a**) the electric and (**b**) magnetic fields at selected wavelengths.

To understand why the absorption is so drastically different, we investigated the field profiles, as shown in Fig. 6. The magnetic field profiles are very different to the first absorber discussed earlier in the manuscript. In particular, there are no strongly confined magnetic fields inside the structures for the insulator phase, or in the gaps for the metallic phase. In both cases, the magnetic field is mostly confined inside the dielectric layer at IR wavelengths. At those wavelengths the size of the structures is sub-wavelength, so the same Mie-like scattering cannot take place in the insulating phase, which leads to a large amount of reflection.

**Figure 7:**
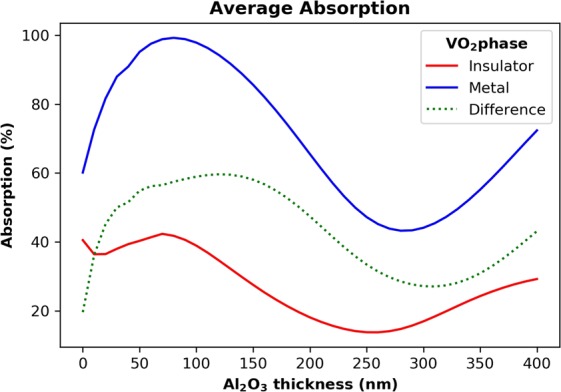
Al_2_O_3_ spacer layer thickness dependence on the absorption for the switchable absorber.

As can be seen in the background plot of Fig. 5, there is a large dip in the absorption around 800 nm. This relates to a large reflectance peak that can be attributed to a Fabry-Perot cavity resonance caused by the 100 nm thick spacer layer creating a metal-insulator-metal structure. This does not occur in the case of the first design, since the structure is too thick. The extinction coefficient of VO_2_ at IR wavelengths in the insulator state is very low, however it increases rapidly in the metallic state, giving rise to the high absorption in the metallic phase, but not in the insulator phase. We investigated the effect of the dielectric spacer layer by varying its thickness from 0 to 400 nm. The results are displayed in Fig. 7. With a spacer thickness of around 50 to 150 nm, we get a high absorption for the metal phase as the peak of the Fabry-Perot cavity induced reflection is outside of the 1,000 to 1,600 nm wavelength range. As the spacer thickness increases, we get multiple reflections from the different layers, inhibiting the performance of the absorber. At around 100 nm, we are near the peak of the difference between the metal and insulator phase absorptions while the metal phase is at a maximum, so that thickness was chosen for this study.

## Conclusions

Two ultra-broadband perfect absorbers made up of arrays of moth-eye structures, using VO_2_ have been designed and investigated. The first, a perfect absorber in both insulating and metallic states from 400 nm to 1,600 nm, is very robust to the incident angle for both TE and TM polarisation, with over 95% absorption up to an incident angle of 60 degrees. The second, a switchable absorber to non-absorber has also been proposed for wavelengths from 1,000 to 1,600 nm, utilising the interesting temperature dependent phase shift in VO_2_. The working mechanism is attributed to a combination of the antireflective nature of the nanostructures due to the broadband impedance matching created by the tapered shape of a parabolic moth-eye type structure, combined with the excitation of localised surface plasmons in the metallic phase and strong confinement of magnetic field by Mie-like resonances in the dielectric case. The Au back reflector should be thick enough (over ~30 nm) to block all transmitted light. While a dielectric spacer thickness of around 100 nm was found to be optimal to allow for ultra-broadband absorption and switchable absorption, with variations in the thickness having negligible impact. The shape of the structure does not necessarily need to be a parabola, although it shows the best performance, since simpler to fabricate structures such as cones and pyramids show comparable ultra-broadband perfect or switchable absorption.

The transition temperature of VO_2_ depends on the growth conditions and composition. Here our VO_2_ sample had a switching temperature of 85 °C, whereas different temperatures of the phase change have been reported elsewhere. This critical temperature could be controlled and lowered to more reasonable, everyday temperatures by doping with other materials^31,32^. This could open up possibilities of active temperature dependent absorbers or tunable color filter^33,34^, for uses such as in smart windows or solar cells at usable daily temperatures.

Although the device is not fabricated in this paper, it is expected that the device could be realised effectively through the nanoimprint method, or even with general electron beam lithography and the corresponding etching processes.

## Methods

The simulations were all performed using the commercially available FDTD solver, Lumerical FDTD solutions. Monitors were placed in the substrate, and behind the source to measure the transmitted and reflected responses respectively. Then the absorption was calculated as A = 1 − T − R, with care taken with regards to the sign of the response of the monitor, since the recorded response is negative from the transmission monitor. Anti-symmetric boundary conditions were used on the x-axis, with symmetric boundary conditions on the y-axis due to the symmetry of the design for TM polarised incident light (and vice versa for TE) to help speed up simulations. Boundary conditions on the z-axis were set to perfectly matched layers (PML). The mesh around the structures was set to 1 nm cells in the x, y and z directions and thorough convergence testing was performed to be sure that the small gaps between the nanostructures did not cause any problems.

## Data Availability

All data generated or analysed during this study is included in this published article.

## References

[1] Azad AK (2016). Metasurface Broadband Solar Absorber. Sci. Rep..

[2] Chandra, S., Franklin, D., Cozart, J., Safaei, A. & Chanda, D. Adaptive Multispectral Infrared Camouflage. *ACS Photonics,* **5 (11)**, 4513–4519 (2018).

[3] Ko, B. Lee, D., Badloe, T. & Rho, J. Metamaterial-based radiative cooling: Towards energy-free all-day cooling. *Energies*, **12**(1), 89 (2019).

[4] Badloe T, Mun J, Rho J (2017). Metasurfaces-Based Absorption and Reflection Control: Perfect Absorbers and Reflectors. J. Nanomater..

[5] Rana AS, Mehmood MQ, Jeong H, Kim I, Rho J (2018). Tungsten-based Ultrathin Absorber for Visible Regime. Sci. Rep..

[6] Kim I, So S, Rana AS, Mehmood MQ, Rho J (2018). Thermally robust ring-shaped chromium perfect absorber of visible light. Nanophotonics.

[7] Li Z, Butun S, Aydin K (2015). Large-Area, Lithography-Free Super Absorbers and Color Filters at Visible Frequencies Using Ultrathin Metallic Films. ACS Photonics.

[8] Cui Y (2014). Plasmonic and metamaterial structures as electromagnetic absorbers: Plasmonic and metamaterial absorbers. Laser Photonics Rev..

[9] Li W (2015). Circularly polarized light detection with hot electrons in chiral plasmonic metamaterials. Nat. Commun..

[10] Wang Z (2016). Circular Dichroism Metamirrors with Near-Perfect Extinction. ACS Photonics.

[11] Kang L (2017). Preserving Spin States upon Reflection: Linear and Nonlinear Responses of a Chiral Meta-Mirror. Nano Lett..

[12] Badloe, T., Kim, I. & Rho, J. Biomimetic ultra-broadband perfect absorbers optimised with reinforcement learning. *Phys. Chem. Chem. Phys*. **22**, 2337–2342 (2020). 10.1039/c9cp05621a 31932814

[13] Franklin D, Frank R, Wu S-T, Chanda D (2017). Actively addressed single pixel full-colour plasmonic display. Nat. Commun..

[14] Tseng ML (2017). Two-Dimensional Active Tuning of an Aluminum Plasmonic Array for Full-Spectrum Response. Nano Lett..

[15] Gholipour B, Zhang J, MacDonald KF, Hewak DW, Zheludev NI (2013). An All-Optical, Non-volatile, Bidirectional, Phase-Change Meta-Switch. Adv. Mater..

[16] Yin X (2015). Active Chiral Plasmonics. Nano Lett..

[17] Tittl A (2015). A Switchable Mid-Infrared Plasmonic Perfect Absorber with Multispectral Thermal Imaging Capability. Adv. Mater..

[18] Qu Y, Li Q, Cai L, Qiu M (2018). Polarization switching of thermal emissions based on plasmonic structures incorporating phase-changing material Ge _2_. Sb. 2 Te 5. Opt. Mater. Express.

[19] Dong W (2019). Wide Bandgap Phase Change Material Tuned Visible Photonics. Adv. Funct. Mater..

[20] Ono M, Chen K, Li W, Fan S (2018). Self-adaptive radiative cooling based on phase change materials. Opt. Express.

[21] Ielmini D, Lacaita AL (2011). Phase change materials in non-volatile storage. Mater. Today.

[22] Raeis-Hosseini N, Rho J (2019). Dual-Functional Nanoscale Devices Using Phase-Change Materials: A Reconfigurable Perfect Absorber with Nonvolatile Resistance-Change Memory Characteristics. Appl. Sci..

[23] Gao T (2017). Three-Dimensional Printed Thermal Regulation Textiles. ACS Nano.

[24] Zhao Y, Huang Q, Cai H, Lin X, Lu Y (2018). A broadband and switchable VO_2_-based perfect absorber at the THz frequency. Opt. Commun..

[25] Liu Z (2017). Design and fabrication of a tunable infrared metamaterial absorber based on VO_2_ films. J. Phys. D Appl. Phys..

[26] Liu Z (2018). Dynamic infrared thin-film absorbers with tunable absorption level based on VO_2_ phase transition. Opt. Mater. Express.

[27] Kocer H (2015). Intensity tunable infrared broadband absorbers based on VO_2_ phase transition using planar layered thin films. Sci. Rep..

[28] Lei L (2019). Tunable and scalable broadband metamaterial absorber involving VO_2_ -based phase transition. Photonics Res..

[29] Weiblen RJ (2016). Optimized moth-eye anti-reflective structures for As_2S_3 chalcogenide optical fibers. Opt. Express.

[30] Contractor R, D’Aguanno G, Menyuk C (2018). Ultra-broadband, polarization-independent, wide-angle absorption in impedance-matched metamaterials with anti-reflective moth-eye surfaces. Opt. Express.

[31] Kim H, Charipar NA, Figueroa J, Bingham NS, Piqué A (2019). Control of metal-insulator transition temperature in VO_2_ thin films grown on RuO_2_ /TiO_2_ templates by strain modification. AIP Adv..

[32] Huang T (2018). Metal-insulator phase transition in Hf-doped VO_2_ (M) thin films: a study on the structural, electrical, optical and infrared radiation properties. Opt. Mater. Express.

[33] Shu F-Z (2018). Dynamic plasmonic color generation based on phase transition of vanadium dioxide. Adv. Opt. Mater..

[34] Lee T, Jang J, Jeong H, Rho J (2018). Plasmonic-and dielectric-based structural coloring: from fundamentals to practical applications. Nano Converg..

